# Prevalence and Antibiogram of *Escherichia coli* and *Staphylococcus* spp. Isolated from Cattle Milk Products Sold in Juja Sub-County, Kenya

**DOI:** 10.1155/2022/5251197

**Published:** 2022-11-21

**Authors:** Julius I. N. Sombie, John Kagira, Naomi Maina

**Affiliations:** ^1^Department of Molecular Biology and Biotechnology, Pan-African University, Institute of Basic Science, Technology and Innovation, P. O. Box 62000-00200, Nairobi, Kenya; ^2^Department of Animal Sciences, Jomo Kenyatta University of Agriculture and Technology, P. O. Box 62000-00200, Nairobi, Kenya; ^3^Department of Biochemistry, Jomo Kenyatta University of Agriculture and Technology, P. O. Box 62000-00200, Nairobi, Kenya

## Abstract

Dairy ruminant milk provides a conducive environment for bacterial proliferation. In animals, these bacteria are exposed to antibiotics, whose overuse has led to increased cases of drug resistance. A cross-sectional study was conducted on milk and milk products vended in Juja Sub-County, Kenya to determine the prevalence of bacteria and antibiogram of *Staphylococcus* spp. and *Escherichia coli*. A total of 169 milk samples were obtained from various outlets in the study area. Milk samples were cultured and isolated bacteria were identified using standard bacteriological procedures. Various bacteria (15 species) were isolated in different proportions. *Staphylococcus* spp. and E. *coli* were isolated from 25.4% and 11.8% of the collected samples, respectively. The highest number of *Staphylococcus* spp. were isolated from raw milk (*n* = 34) while the highest number of E. *coli* where isolated from fermented milk (*n* = 15). *Staphylococcus* spp. and E. *coli* isolates were subjected to antimicrobial susceptibility tests using CLSI guidelines. The *Staphylococcus* spp. isolates were highly resistant to penicillin G (93%) but susceptible to norfloxacin (100%), gentamicin (90.6%), and chloramphenicol (86%). The E. *coli* isolates were highly resistant to cephalexin (85%) and ceftazidime (60%) but susceptible to chloramphenicol (100%), norfloxacin (95%), gentamicin (95%), azithromycin (95%) and cefepime (80%). Furthermore, 44.3% of *Staphylococcus* spp. and 50% of *E. coli* isolates had a Multiple Antibiotic Resistance (MAR) Index greater than 0.2. This implies that these bacteria were high-risk bacteria whose treatment with current antibiotics would be challenging. The high prevalence and multidrug resistance patterns shown by the *Staphylococcus* spp. and E. *coli* isolated from milk products in Juja Sub-county highlights the importance of proper handling and processing of milk from the farm to consumers. This will in turn reduce the possibility of zoonotic transfer of multidrug-resistant bacteria.

## 1. Introduction

The high nutritional content of dairy ruminant milk makes it conducive to bacterial growth. Bacteria, such as *Escherichia coli* and *Staphylococcus* spp., can colonize the udder of cattle leading to the occurrence of mastitis [1, 2]. These bacteria are transmitted to the dairy animals from the environment, during the milking process when the milker's hands or milking equipment lack adequate hygiene or along the milk product production chain by contaminated water or utensils used by vendors [3]. Humans can subsequently consume these bacteria leading to disease.

The bacterium E. *coli* is of great importance in milk safety and health and has been labeled as an indicator of fecal contamination, inadequate sanitary measures, and handling practices in milk [4]. This bacterium is one of the most common causes of intestinal tract infections and diarrheal diseases [5] making it the second most causative agent of child death globally [6]. The emergence and spread of antibiotic resistance has compounded the morbidity caused by E. *coli*. *Staphylococcus* spp. is among the most important pathogens affecting human health with staphylococcal poisoning being the most common type of food-borne disease [7]. Although *Staphylococcus* spp. from dairy products are nonpathogenic they can become opportunistic and cause nosocomial infections [8].

Because of the aggressive use and misuse of antibiotics, we have established that bacteria such as *Staphylococci* spp. and E. *coli* are becoming increasingly antibiotic-resistant [9, 10]. For example, studies undertaken in Kenya reported multidrug resistance ranging from 18% to 34% in *Staphylococcus* spp. isolated from milk from cows [11]. It also reported raw milk samples from Ethiopia to have multidrug-resistant E. *coli* [12]. These resistant bacteria can be transferred to humans through milk products that have been contaminated, adulterated, or unpasteurized [13].

Africa may be taking a heavier toll of antimicrobial resistance (AMR) than the rest of the world because surveillance data are scarce and some reports only give a fraction of the real situation [14]. According to the African Union Framework for Antimicrobial Resistance Control (2020–2025) in the efforts to combat antimicrobial resistance, one of the primary goals is to increase the number of foods tested for pathogens and their resistance patterns. The current study sought to evaluate the bacteria present in milk and the antimicrobial patterns of *Staphylococcus* spp. and E. *coli* isolated from raw milk and milk products sold in Juja Sub-county, Kenya. The area of study is characterized by urban and peri-urban keeping of dairy cattle.

## 2. Study Area

We carried this study out in 5 wards of Juja Sub-County (Juja, Withethie, Murera, Kalimoni, and Theta) of Kiambu County, Kenya. Juja Sub-County is located 30 kilometers north of Nairobi with geographical coordinates of 1° 11′ 0″ south and 37° 7′ 0″ east. The study area has 300,948 inhabitants with an average household size of 2.9. It has a land area of 342 sq. km [15] and is 1519m above sea level with a climate classified as warm and temperate. Furthermore, it has an average temperature of 18.8°C. Juja Sub-County has about 1,457 households rearing 5,084 exotic dairy cattle and 1,148 households rearing 9,417 indigenous cattle [16]. In Juja Sub-County, there is a rising human population whose milk needs are partly met by small-scale dairy peri-urban farming.

### 2.1. Collection of Milk Samples

The current study used the cross-sectional design to collect samples of milk products from the 5 wards of Juja Sub-County. The sample size was determined using the formula adjusted for small populations [17].

(1) $$\begin{array}{c} n=\frac{1.96^{2}\times P_{\exp}\left(1-P_{\exp}\right)}{d^{2}}\;and\;n_{adj}=\frac{N\times n}{N+n}\text{ .} \end{array}$$

Using these formulae, the minimum sample size was 150. The study planned to collect equal number of samples but the availability of different milk types led to differences. In the current study, we collected a total of 169 samples of cattle milk products from different outlets and manufacturers on the main roads and markets of the wards (Table 1). The UHT-packed milk sampled included 2 different brands of milk most sold within the 5 wards.

### 2.2. Morphological Characterization of Bacteria

We inoculated the milk samples onto Sheep Blood Agar (HiMedia, India) and MacConkey Agar (Oxoid, UK). Briefly, 100 *μ*l of the milk samples were inoculated onto 5% Sheep Blood Agar and MacConkey Agar. For *Staphylococcus* spp. and E. *coli*, we cultured isolates as described previously [18, 19]. For *Staphylococcus* spp. discrete colonies from Sheep Blood Agar were subcultured unto Mannitol Salt Agar (Oxoid, UK). Colonies that appeared pink or fermented mannitol producing a pH that changed the color of the Mannitol Salt Agar from pink to yellow were presumptively taken to be *Staphylococcus* spp. [20]. For E. *coli*, discrete bright pink colonies from MacConkey Agar were subcultured onto Eosin Methylene Blue Agar (Oxoid, UK). It presumptively took colonies producing a green metallic sheen with a dark nucleated center to be E. *coli* colonies (20). We incubated aerobically all plates at 37°C for 18–20 hours.

### 2.3. Identification of Bacteria Using MALDI-TOF

All isolated bacteria and presumptive *Staphylococcus* spp. and E. *coli* bacteria were further identified by Matrix-assisted Laser Desorption Ionization-time of Flight Mass Spectrometry **(**MALDI-TOF) using procedures described [21] with slight modifications. Briefly, discrete overnight colonies of bacteria isolated from the milk samples were picked from petri dishes using sterile pipette tips and added to 0.5 *μ*l 25% formic acid. This mixture was then smeared on a FlexiMass-DS^TM^ disposable polymeric plate and 0.5 *μ*l of the matrix, *α*-cyano-4-hydroxycinnamic acid (CHCA) was added, and the plate inserted into the MALDI-TOF equipment. The bacteria colonies were analyzed using the iDplus Axima Confidence Reflectron (SHIMADZU BIOTECH, Japan) in a positive linear mode (2,000–20,000 m/z range). The spectra profiles of each bacterium, generated by detection of proteinaceous parts by the MALDI-TOF equipment, serve as a unique fingerprint for each bacteria and were searched against the SuperSpectra in the Spectral ARchive And Microbial Identification System (SARAMIS) database for bacterial identification.

### 2.4. Antibiotic Susceptibility Test

The antibiotic susceptibility test for isolated *Staphylococcus* spp. and E. *coli* bacteria was conducted according to CLSI guidelines [22]. For *Staphylococcus* spp., their susceptibility to 7 antibiotics (penicillin G, gentamicin, azithromycin, erythromycin, tetracycline, chloramphenicol, and norfloxacin) was determined. For E. *coli* bacteria, their susceptibility to 12 antibiotics (ampicillin, amoxicillin-clavulanate, cephalexin, cefoxitin ceftazidime, cefepime, gentamicin, streptomycin, azithromycin, tetracycline, chloramphenicol, and norfloxacin) was determined. We purchased all the antibiotics from Oxoid (Oxoid, UK). *Escherichia coli* ATCC 25922 and S. *aureus* ATCC 25923 obtained from the Kenya Medical Research Institute were used as reference strains.

### 2.5. Determination of MAR Index

The Multiple Antibiotic Resistance (MAR) Index was calculated using the following formula:

(2) $$\begin{array}{c} MAR\;index=\frac{a}{b}\text{,} \end{array}$$

where “*a*” is the total number of antibiotics to which a particular bacterium is resistant and “*b*” is the total number of antibiotics against which the bacterium was tested [23].

### 2.6. Data Analysis

We entered all data into MS Excel (Microsoft, USA) and using SPSS 28 (Microsoft, USA), computed all frequencies and percentages using descriptive statistics. We analyzed the difference in prevalence of bacteria in milk products and wards using Friedman test followed by Wilcoxon signed-rank tests with Bonferroni adjustment. We set statistical significance level at 0.05 (*p* < 0.05).

## 3. Results

### 3.1. Prevalence of Bacteria in Various Milk and Milk Products

Based on the various proteins, serving as structural and functional parts in the isolated bacteria, various unique spectra were generated by the MALDI-TOF equipment leading to the identification of 15 species of bacteria with varying prevalence from 130 (76.9%) of the 169 collected milk samples (Table 2). The most common bacteria isolated were S. *sciuri*, E. *coli, Enterobacter cloaceae, Serratia marcescens,* S. *saprophyticus,* and *Enterobacter asburiae*. The milk type with the lowest bacterial prevalence was UHT-packed milk (6.7%) followed by yogurt (8.3%). There was a statistically significant difference (*χ*^2^(4) = 45.916, *p* < 0.001) in the prevalence of bacteria among the milk products. Raw milk had a significantly higher (*p* < 0.001) proportion of bacteria compared to yogurt, UHT-packed milk, and pasteurized milk.

### 3.2. Prevalence of Bacteria in Milk and Milk Products Obtained from Various Wards

The prevalence of bacteria isolated from milk from the 5 wards of Juja Sub-County are shown in Table 3. In descending order, most of the bacterial isolates were obtained in milk products sampled from Murera, followed by Juja, Theta, and Kalimoni wards, and the least were from Withethie ward. There was no statistically significant difference in the prevalence of bacteria as related to ward of origin of milk from specific wards (*χ*^2^(4) = 7.914, *p* < 0.095)

### 3.3. Resistance of Isolated *Staphylococcus* spp

The *Staphylococcus* spp. isolates showed high resistance to penicillin G (93%) but were highly susceptible to norfloxacin, gentamicin, and chloramphenicol (Table 4). Only 2 isolates (S. *saprophyticus* and S. *warneri*) from UHT-packed milk were susceptible to all antibiotics. The resistance pattern of the isolated *Staphylococcus* spp., shown in Table 5 revealed that most of the isolates were resistant to penicillin G. The resistant pattern further showed that 92.8% (*n* = 13) of the isolated S. *saprophyticus* were resistant only to penicillin. The only isolated S. *xylosus* bacterium was resistant only to penicillin, gentamicin, erythromycin, azithromycin, and tetracycline. Furthermore, the MAR index of the *Staphylococcus* spp. revealed that 44.3% (*n* = 10) of the isolated *Staphylococcus* spp. had a MAR greater than 0.2.

### 3.4. Antibiogram of Isolated *Escherichia coli*

The antibiogram of E. *coli* isolates from milk and milk products in Juja Sub-County are shown in Table 6. The E. *coli* isolates indicated the highest resistance to Cephalexin (85%) and the highest susceptibility to Chloramphenicol (100%). The resistance pattern of the E. *coli* isolates (Table 7) showed that 95% (19/20) were resistant to at least one antibiotic. The antibiotic against which the most resistance was demonstrated was cephalexin (85%), followed by cefoxitin (50%). Only one E. *coli* isolate was susceptible to all 12 antibiotics. We observed that there was an overall decrease in resistance against cephalosporins as the generation of the cephalosporins increased. Furthermore, the MAR index of the E. *coli* isolates revealed that 50% (*n* = 10) had a MAR index greater than 0.2.

## 4. Discussion

We geared the current study toward investigating the prevalence of bacteria and the antibiogram of *Staphylococcus* spp. and E. *coli* bacteria isolated from milk and milk products sold in various outlets in Juja Sub-County, Kenya. The products are sold to an urban set-up with high human population. Various bodies such as the African Union and Food and Agricultural Organization (FAO) have called for surveillance studies on the occurrence of bacteria in milk, leading to contamination and emergence of AMR in different set-ups to develop intervention strategies [24, 25].

The current study showed that high proportion of the milk samples was contaminated with different bacteria. It was seen that raw milk had the highest proportion of bacteria. Bacteria contaminated the raw milk either during the milking process, handling by retailers, or storage under poor conditions. This high percentage of raw milk samples contaminated with bacteria is alarming. In a recent study in urban and peri-urban centers in Kenya, 86% of milk was sold as raw [26]. Milk hawkers in this channel of marketing seldomly cool or preserve the milk after collection from dairy farmers. Since farmers rarely observe the antibiotic withdrawal periods, the milk could also have bacteria resistant to various antibiotics used to treat mastitis and other animal diseases. Apart from Nairobi county, raw milk is the most consumed milk type by both rural and urban consumers in Kenya [27]. One main reason for this preference is the low price of raw milk, which is 60–70% cheaper compared to pasteurized milk [28]. A study carried out in Tanzania and Kenya showed consumers prefer raw milk to UHT-packed milk, as they consider the latter to contain additives [29]. Others prefer raw milk as they can buy the amount of milk they need, as opposed to UHT-packed milk, which is sold relatively in small standard sizes. The use of pasteurized milk is however recommended since it has reduced bacterial load levels [30] as seen in the present study.

The current study showed that only a few pasteurized milk samples had E. *coli,* similar to a study from Iran that recorded E. *coli* in only 9% of pasteurized dairy milk samples [31]. This, however, is in contrast to a higher prevalence of milk sampled from similar environments in Brazil. In that study, an E. *coli* prevalence of 41.1% in pasteurized milk was reported. Furthermore, in Ghana, the E. *coli* prevalence in pasteurized milk was observed at 78% [32]. A reason for this difference in prevalence may be that in the present study, pasteurized milk was sold from milk dispensing machines, while in the latter study, pasteurized milk was sold in open market containers.

In the current study, most of the E. *coli* isolates were isolated from fermented milk (Mala). Mala milk is prepared by fermenting in local calabashes [33]. Thus, the high percentage of E. *coli* isolated from this milk type may be due to the poor handling techniques of the milk during the preparation process. Furthermore, E. *coli* bacteria serves as an indicator of poor hygiene practices or even fecal contamination [34]. People in Kenya, who tend to consume more fermented milk products such as Mala, may be at risk.

Both *Staphylococcus* spp. and E. *coli* were not isolated from sampled yogurt products. Similarly, in other studies in Iran and Burkina Faso, low or no E. *coli* were isolated from yogurt samples [35, 36]. A reason for the low recovery from yogurt is that the lactic acid bacteria that are used as starter culture in the production of yogurt have antibacterial effects and properties against E. *coli* [37].

In the current study, *Staphylococcus* spp. were most resistant to penicillin G. Locally, 100% of the *Staphylococcus* spp. isolated from milk from dairy goats in Juja and Thika Sub-Counties were shown to be resistant to penicillin G [10]. The high rate of resistance to penicillin G is consistent with a study of *Staphylococcus* spp. in artisanal dairy products in Benin where 100% of *Staphylococcus* spp. were resistant to penicillin G [38]. Furthermore, *Staphylococcus* spp. in the current study were highly susceptible to chloramphenicol, which agrees with a study of similar bacteria from unpasteurized milk in South Africa, which had a susceptibility to chloramphenicol at 93.7%. The *Staphylococcus* spp. in the current study had low resistance to gentamicin (7%) and norfloxacin (0%). Similarly, in the only study describing mastitis in Juja Sub-County [39], the *Staphylococcus* spp. from cow milk had a 6% resistance against Gentamicin. In addition, the current study agrees with the study on raw milk from Kenya where *Staphylococcus* spp. had a 4% resistance to fluoroquinolones and 5.4% resistance to gentamicin [11]. With these low percentages, we can postulate that bacteria from the study area are susceptible to norfloxacin and gentamicin because these antibiotics are possibly not frequently used to treat infections in dairy cattle in this area. The E. *coli* isolates in the current study were highly susceptible to chloramphenicol, also similar to the study by [39], norfloxacin, gentamicin, and azithromycin. These results are similar to a study of raw milk sold in markets in Egypt [40]. The current study reports resistance of the E. *coli* isolates to the older generations of cephalosporins. These *β*-lactam antibiotics are among the antibiotics used to treat Mastitis in Kenya [41] since few effective alternatives of antibiotics exist for mastitis treatment.

MAR index has proven to be a low-cost method for determining a high-risk source of infection from bacteria where particular antibiotics are frequently used [42]. The current study showed that 44.3% of the *Staphylococcus* spp. and half of the E. *coli* isolates had a MAR index greater than 0.2 indicating these isolates were having high multiple drug resistance making them high-risk bacteria. Resistance could have emerged from cattle that have been repeatedly treated with antibiotics and thus gained multidrug-resistant bacteria that could be transmitted to humans. This is a call for concern as 66.2% and 55% of Kenyans consume raw milk at least once a day in rural and urban households, respectively [33].

The high burden of AMR is evident in humans, where a review by O'Niell [43] forecasted that there will be about 4 million human deaths in Africa attributed to antimicrobial-resistant bacteria by the year 2050. The current study showed that all the isolated staphylococci bacteria were CoNS, some of which were multidrug-resistant. Multidrug-resistant*Staphylococcus* spp. have also emerged and are becoming a public problem on a global scale [44], some reasons being the fact that they have a short incubation time and are capable of producing toxins [45]. *Staphylococcus* spp., especially those that have developed antimicrobial resistance, can be transferred to humans through milk products that have been contaminated, adulterated, or unpasteurized [13]. *Staphylococcus* spp. have been found to occur in the mammary glands of cattle and can easily pass on to milk and milk products used for human consumption [2]. In their study [39], *Staphylococcus* spp. was the most prevalent bacteria (56.7%) isolated from milk from cows with subclinical mastitis in the same study area as the current study. The four species of staphylococci from the current study, S. *saprophyticus,* S. *warneri*, S. *xylosus,* and S. *sciuri*, have all been listed among causative agents of orthopedic infections in humans [46]. Furthermore, food poisoning due to dairy product contamination by CoNS has been reported in Morocco [47] with food poisoning outbreaks due to CoNS reported in Brazil [48].

Coliforms were also isolated from milk and milk products sold at various outlets in Juja Sub-County, Kenya. These included E. *coli*, C. *freundii*, K. *pneumoniae*, E. *cloacae,* E. *asburiae,* and *Serratia marcescens*. The presence of these coliforms may point to low sanitary conditions of the vended milk and pose potential risks to consumers of the milk products from which these coliforms were isolated [49]. It has been observed that E. *coli* isolated from dairy raw milk can also affect the health of human beings through the production of toxins [18] as seen in Shiga-toxin producing E. *coli* which produces verocytotoxins that cause severe diseases in humans such as hemorrhagic colitis [50] or life-threatening complications including kidney damage and renal failure [51].

## 5. Conclusion

The present study showed that both *Staphylococcus* spp. and E. *coli* were among the bacteria prevalent in milk sold in Juja Sub-County, Kenya, with the raw milk being the most contaminated. Since these bacteria are highly prevalent where good hygiene or proper handling is lacking, street vendors and milk farmers alike should be trained on strategies to improve sanitary measures in milk handling. Similar to other studies in the locality, most of the *Staphylococcus* spp. isolates from the milk products were resistant to penicillin G but all were susceptible to norfloxacin. Thus, as observed in the study by others [10], penicillin G is currently an unsuitable drug for mastitis treatment in the area while norfloxacin or gentamicin could be better drugs for treatment of mastitis caused by *Staphylococcus* spp. as seen in the subclinical mastitis study by [39] in Juja Sub-County, Kenya. For the E. *coli* isolated from the dairy products, the most efficacious drugs included gentamicin, azithromycin, norfloxacin, and chloramphenicol. However, resistance against cephalosporins was quite notable making them not-so-good drug candidates against mastitis caused by E. *coli* in the study area. In the same study area as the current study, Kagira et al. [39] reported a 43.3% prevalence of subclinical mastitis in udders of dairy cows, meaning possible antibiotic intervention by farmers. More studies are needed to ascertain the antibiotic types used by farmers in the study area and Kenya at large so that AMR in Kenya can be easily mapped. Such information will inform policies regarding the control of AMR. The current study highlights the importance of bacteria present in milk and milk products in the study area and emphasizes the need for proper handling and processing of milk and the possibility of transmission of these resistant bacteria to human beings. Studies should be undertaken in the study area to determine the potential of antibiotic-resistant bacteria crossing the animal-human barrier, causing hard-to-treat infections in humans.

## Figures and Tables

**Table 1:** Milk products sampled in Juja Sub-County, Kenya (*n* = 169).

Ward	Milk products				
	Raw milk	Pasteurized milk	UHT-packed milk	Locally prepared yogurt	Fermented milk
Juja (*n* = 45)	15	6	6	6	12
Withethie (*n* = 20)	9	0	6	2	3
Murera (*n* = 43)	29	2	6	4	2
Kalimoni (*n* = 23)	12	1	6	0	4
Theta (*n* = 38)	30	0	6	0	2
Total	95	9	30	12	23

UHT = ultra heat treated.

**Table 2:** Prevalence of various bacteria isolated from milk and milk products sold in various outlets in Juja Sub-County, Kenya.

Bacteria isolated	Type of milk and milk product					
	Raw milk (*n* = 95)	Pasteurized milk (*n* = 9)	UHT-packed milk (*n* = 30)	Yoghurt (*n* = 12)	Fermented milk (*n* = 23)	Total (*n* = 169)
S. s*ciuri*	17 (17.8%)	4 (44.5%)	0 (0)	0 (0)	0 (0)	21 (12.4%)
S. *saprophyticus*	10 (10.5%)	2 (22.2%)	1 (3.3%)	0 (0%)	1 (4.4%)	14 (8.3%)
S. *warneri*	6 (6.3%)	0 (0%)	1 (3.3%)	0 (0%)	0 (0%)	7 (4.1%)
S. *xylosus*	1 (1.1%)	0 (0%)	0 (0%)	0 (0%)	0 (0%)	1 (0.6%)
E. *coli*	4 (4.2%)	1 (11.1%)	0 (0%)	0 (0%)	15 (65.2%)	20 (11.8%)
*Serratia marcescens*	15 (15.8%)	0 (0%)	0 (0%)	0 (0%)	0 (0%)	15 (8.9%)
*Enterobacter asburiae*	10 (10.5%)	0 (0%)	0 (0%)	0 (0%)	0 (0%)	10 (5.9%)
*Enterobacter cloacae*	9 (9.5%)	2 (22.2%)	0 (0%)	1 (8.3%)	7 (30.4)	19 (11.3%)
*Bacillus cereus*	6 (6.3%)	0 (0%)	0 (0%)	0 (0%)	0 (0%)	6 (3.6%)
*Klebsiella pneumoniae*	5 (5.3%)	0 (0%)	0 (0%)	0 (0%)	0 (0%)	5 (2.9%)
*Aeromonas* spp.	4 (4.2%)	0 (0%)	0 (0%)	0 (0%)	0 (0%)	4 (2.4%)
*Citrobacter freundii*	3 (3.2%)	0 (0%)	0 (0%)	0 (0%)	0 (0%)	3 (1.8%)
*Exiguobacterium acetylicum*	2 (2.1%)	0 (0%)	0 (0%)	0 (0%)	0 (0%)	2 (1.2%)
*Acinetobacter baumanii*	2 (2.1%)	0 (0%)	0 (0%)	0 (0%)	0 (0%)	2 (1.2%)
*Raoultella ornithinolytica*	1 (1.1%)	0 (0%)	0 (0%)	0 (0%)	0 (0%)	1 (0.6%)
Total	95 (100%)	9 (100%)	2 (6.7%)	1 (8.3%)	23 (100%)	130 (76.9%)

**Table 3:** Prevalence of bacteria isolated from milk and milk products sold in various outlets in wards of Juja Sub-County, Kenya.

Bacteria	Juja (*n* = 45)	Withethie (*n* = 20)	Murera (*n* = 43)	Kalimoni (*n* = 23)	Theta (*n* = 38)	Total (*n* = 169)
S. *sciuri*	3 (6.7%)	2 (10%)	5 (11.6%)	4 (17.4%)	7 (18.4%)	21 (12.4%)
S. *saprophyticus*	2 (4.5%)	4 (20%)	2 (4.7%)	2 (8.7%)	4 (10.5%)	14 (8.3%)
S. *warneri*	2 (4.5%)	1 (5%)	2 (4.7%)	0 (0%)	2 (5.3%)	7 (4.1%)
S. *xylosus*	1 (2.2%)	0 (0%)	0 (0%)	0 (0%)	0 (0%)	1 (0.6%)
*Escherichia coli*	12 (26.7%)	4 (20%)	0 (0%)	1 (4.3%)	3 (7.9%)	20 (11.8%)
*Serratia marcescens*	4 (8.9%)	0 (0%)	4 (9.3%)	2 (8.7%)	5 (13.2%)	15 (8.9%)
*Enterobacter asburiae*	1 (2.2%)	1 (5%)	4 (9.3%)	1 (4.3%)	3 (7.9%)	10 (5.9%)
*Enterobacter cloacae*	3 (6.7%)	0 (0%)	8 (18.6%)	1 (4.3%)	7 (18.4%)	19 (11.3%)
*Bacillus cereus*	2 (4.5%)	1 (5%)	3 (6.9%)	0 (0)	0 (0%)	6 (3.6%)
*Klebsiella pneumoniae*	2 (4.5%)	0 (0%)	3 (6.9%)	0 (0)	0 (0%)	5 (2.9%)
*Aeromonas* spp.	0 (0%)	0 (0%)	0 (0%)	4 (17.4%)	0 (0%)	4 (2.4%)
*Citrobacter fruendii*	1 (2.2%)	0 (0%)	0 (0%)	2 (8.7%)	0 (0%)	3 (1.8%)
*Exiguobacterium acetylicum*	0 (0%)	0 (0%)	1 (2.3%)	0 (0%)	1 (2.6%)	2 (1.2%)
*Acinetobacter baumanii*	0 (0%)	0 (0%)	2 (4.7%)	0 (0%)	0 (0%)	2 (1.2%)
*Raoultella ornithinolytica*	0 (0%)	1 (5%)	0 (0%)	0 (0%)	0 (0%)	1 (0.6%)
Total	33 (73.6%)	14 (70%)	34 (79%)	17 (73.8%)	32 (24.61)	130 (76.9%)

**Table 4:** Antibiotic resistance of *Staphylococcus* spp. (*n* = 43) isolated from milk and milk products sold in various outlets in Juja Sub-County, Kenya.

Antibiotic (*μ*g/disc)	S. *sciuri*(*n* = 21) (%)	S.*saprophyticus*(*n* = 14) (%)	S. *warneri*(*n* = 7) (%)	S. *xylosus*(*n* = 1) (%)
Penicillin G (10 IU)	100	92.8	85.7	100
Gentamicin (10 *μ*g)	9.5	0	0	100
Erythromycin (15 *μ*g)	52.3	0	42.8	100
Azithromycin (15 *μ*g)	38.1	0	28.5	100
Tetracycline (30 *μ*g)	33.3	0	28.5	100
Norfloxacin (10 *μ*g)	0	0	0	0
Chloramphenicol (30 *μ*g)	4.8	0	28.5	0

**Table 5:** Antibiotic resistance pattern showed by *Staphylococcus* spp. isolated from milk and milk products from various outlets in Juja Sub-County, Kenya.

Number of antibiotics resistant to	Antibiotic resistance pattern	No. of isolates (%)	
		S. *sciuri*	S. *warneri*
0	—	0 (0%)	1 (14.2%)

1	P	6 (28.5%)	1 (14.2%)

2	P and GEN	2 (9.5%)	2 (29%)
	P and E	2 (9.5%)	0 (0%)
	P and TE	1 (4.7%)	1 (14.2%)
	P and C	0 (0%)	0 (0%)

3	P, E and AZT	4 (19.1%)	1 (14.2%)
	P, E and TE	1 (4.7%)	0 (0%)

4	P, E, AZT and TE	4 (19.1%)	0 (0%)

5	P, GEN, E, AZT and TE	0 (0%)	1 (14.2%)
	P, E, AZT, TE and C	1 (4.7%)	0 (0%)

P = Penicillin G, E = Erythromycin, GEN = Gentamicin, C=Chloramphenicol, AZT = Azithromycin, TE = Tetracycline.

**Table 6:** Antibiogram of E. *coli* (*n* = 20) isolated from milk and milk products sold in various outlets in Juja Sub-County, Kenya.

Antibiotic (concentration)	Resistant (%)	Intermediate (%)	Susceptible (%)
Gentamicin (10 *μ*g)	5	0	95
Cephalexin (30 *μ*g)	85	15	0
Cefoxitin (30 *μ*g)	55	0	45
Ceftazidime (30 *μ*g)	60	10	30
Cefepime (30 *μ*g)	15	5	80
Ampicillin (10 *μ*g)	30	50	20
Azithromycin (10 *μ*g)	5	0	95
Amoxicillin-clavulanate (20/10 *μ*g)	35	15	50
Streptomycin (10 *μ*g)	50	0	50
Tetracycline (30 *μ*g)	25	5	70
Chloramphenicol (30 *μ*g)	0	0	100
Norfloxacin (10 *μ*g)	0	5	95

**Table 7:** Antibiotic resistance pattern showed by E. *coli*. isolated from milk and milk products from various outlets in Juja Sub-County, Kenya.

Number of antibiotics resistant to	Antibiotic resistance pattern	No. of isolates (%)
0	—	1 (5%)

1	CN	3 (15%)
	FOX	1 (5%)

2	CN and FOX	2 (10%)
	CN and CTZ	1 (5%)
	CTZ and S	1 (5%)

3	CN, CTZ and TE	1 (5%)
	CN, FOX and CTZ	1 (5%)

4	CN, FOX, CTZ and AMP	1 (5%)
	CN, CTZ, AMC and S	1 (5%)

5	CN, CTZ, AMP, AMC and S	1 (5%)
	CN, FOX, CTZ, CPM and AMP	1 (5%)
	CN, FOX, AMP, S and TE	1 (5%)

6	CN, FOX, CTZ, CPM, AMC and S	1 (5%)
	CN, FOX, CTZ, AMC, S and TE	1 (5%)

7	CN, FOX, CTZ, AMP, AMC, S and TE	1 (5%)

8	CN, G, FOX, CTZ, CPM, AMP, AZT, AMC, S and TE	1 (5%)

CN = Cephalexin, FOX = Cefoxitin, CTZ = Ceftazidime, CPM = Cefepime, S =Streptomycin, AMP = Ampicillin, AZT = Azithromycin, AMC = Amoxicillin-clavulanate, TE = Tetracycline, G = Gentamicin.

## Data Availability

The raw data used to support the findings of this study are available from the corresponding author upon request.

## References

[1] Burvenich C., van Merris V., Mehrzad J., Diez-Fraile A., Duchateau L. (2003). Severity of E. *coli* mastitis is mainly determined by cow factors. *Veterinary Research*.

[2] Sasidharan S., Prema B., Yoga Latha L. (2011). Antimicrobial drug resistance of *Staphylococcus aureus* in dairy products. *Asian Pacific Journal of Tropical Biomedicine*.

[3] Silva J. G., Camargo A. C., Melo R. P. B. (2022). A positive staphylococcus spp. in bovine mastitis, milkers, milking environment, and the circulation of different MRSA clones at dairy cows farms in the northeast region of Brazil. *Ciência Rural*.

[4] Price R. G., Wildeboer D. (2017). E. *coli* as an indicator of contamination and health risk in environmental waters. *Escherichia Coli-Recent Advances on Physiology, Pathogenesis and Biotechnological Applications*.

[5] Gwimbi P., George M., Ramphalile M. (2019). Bacterial contamination of drinking water sources in rural villages of mohale basin, Lesotho: exposures through neighbourhood sanitation and hygiene practices. *Environmental Health and Preventive Medicine*.

[6] Elafify M., Khalifa H. O., Al-Ashmawy M. (2020). Prevalence and antimicrobial resistance of shiga toxin-producing*Escherichia coli* in milk and dairy products in Egypt. *Journal of Environmental Science and Health, Part B*.

[7] Hennekinne J. A., De Buyser M. L., Dragacci S. (2012). *Staphylococcus aureus* and its food poisoning toxins: characterization and outbreak investigation. *FEMS Microbiology Reviews*.

[8] Irlinger F. (2008). Safety assessment of dairy microorganisms: coagulase-negative*Staphylococci*. *International Journal of Food Microbiology*.

[9] Okoko I. M., Kagira J., Kiboi D., Maina N. (2021). Occurrence of beta-lactamases genes in beta-lactam resistant bacteria isolated from milk of goats with sub-clinical mastitis in Thika sub-county Kenya. *World’s Veterinary Journal*.

[10] Mahlangu P., Maina N., Kagira J. (2018). Prevalence, risk factors, and antibiogram of bacteria isolated from milk of goats with subclinical mastitis in Thika east sub-county, Kenya. *Journal of Veterinary Medicine A*.

[11] Mbindyo C. M., Gitao G. C., Plummer P. J., Kulohoma B. W., Mulei C. M., Bett R. (2021). Antimicrobial resistance profiles and genes of staphylococci isolated from mastitic cow’s milk in Kenya. *Antibiotics*.

[12] Tadesse H. A., Gidey N. B., Workelule K. (2018). Antimicrobial resistance profile of E. *coli* isolated from raw cow milk and fresh fruit juice in Mekelle, Tigray, Ethiopia. *Veterinary Medicine International*.

[13] Yener F. Y. G., Korel F., Yemenicioğlu A. (2009). Antimicrobial activity of lactoperoxidase system incorporated into cross-linked alginate films. *Journal of Food Science*.

[14] Alabi A. S., Frielinghaus L., Kaba H. (2013). Retrospective analysis of antimicrobial resistance and bacterial spectrum of infection in Gabon, Central Africa. *BMC Infectious Diseases*.

[15] Kenya National Bureau of Statistics (2019). Population by county and sub-county.

[16] Kenya National Bureau of Statistics (2019). Kenya population and housing census.

[17] Thrusfield M. (2018). *Veterinary Epidemiology*.

[18] Yohannes G. (2018). Isolation, identification and antimicrobial susceptibility testing of *Escherichia coli* isolated from selected dairy farms in and around Mekelle, Ethiopia. *Journal of Dairy, Veterinary & Animal Research*.

[19] Jahan M., Rahman M., Parvej M. S. (2015). Isolation and characterization of *Staphylococcus aureus* from raw cow milk in Bangladesh. *Journal of Advanced Veterinary and Animal Research*.

[20] Markey F. L., Archambault M., Cullinane A., Maguire D. (2013). *Clinical Veterinary Microbiology*.

[21] Nsofor A., Nsofor C. M., Ukwandu N. C. D., Kaasch A. J. (2021). MALDI-TOF MS based identification and characterization of staphylococci isolates from human and animal sources. *Tropical Journal of Natural Product Research*.

[22] CLSI Performance Standards for Antimicrobial Susceptibility Testing (2020). *Thirtieth Information Supplement*.

[23] Osundiya O., Oladele R., Oduyebo O. (2013). Multiple antibiotic resistance (MAR) indices of Pseudomonas and *Klebsiella species* isolates in lagos university teaching hospital. *African Journal of Clinical and Experimental Microbiology*.

[24] (2021). *African Union Framework for Antimicrobial Resistance Control 2020–2025*.

[25] (2021). *The FAO Action Plan on Antimicrobial Resistance 2021–2025*.

[26] Jein M. A. R., Naukkarinen M., Rasmussen T. O. (2015). *Smallholder Dairy Farmers Challenges in Milk Production and Marketing in Bathi*.

[27] Mtimet Mand N., Karugia J. (2020). *Consumers Perception of Milk Safety in Kenya*.

[28] Karanja A. M. (2003). *The Dairy Industry in Kenya: The Post-Liberalization Agenda*.

[29] Galié A., Farnworth C. R., Njiru N., Alonso S. (2021). Intra-household handling and consumption dynamics of milk in peri-urban informal markets in Tanzania and Kenya: a gender lens. *Sustainability*.

[30] Popovic-Vranjes A., Popović M., Jevtić M. (2015). Raw milk consumption and health. *Srpski arhiv za celokupno lekarstvo*.

[31] Vahedi M., Nasrolahei M., Sharif M., Mirabi A. M. (2013). Bacteriological study of raw and unexpired pasteurized cow’s milk collected at the dairy farms and supermarkets in Sari city in 2011. *Journal of Preventive Medicine and Hygiene*.

[32] Adzitey F., Amposah C., Teye G. (2019). Prevalence and antimicrobial resistance patterns of E. *coli* isolates from cow milk, milk products and handlers in the tamale metropolis of Ghana. *Nigerian Veterinary Journal*.

[33] Njarui D. M. G., Gatheru M., Wambua J. M., Nguluu S. N., Mwangi D. M., Keya G. A. (2011). Consumption patterns and preference of milk and milk products among rural and urban consumers in semi-arid Kenya. *Ecology of Food and Nutrition*.

[34] Thaker H. C., Brahmbhatt M. N., Nayak J. B. (2012). Study on occurrence and antibiogram pattern of *Escherichia coli* from raw milk samples in Anand, Gujarat, India. *Veterinary World*.

[35] Rahimi E., Chaleshtori S. S., Parsaei P. (2011). Prevalence and antimicrobial resistance of *Escherichia coli* O157 isolated from traditional cheese, ice cream and yoghurt in Iran. *African Journal of Microbiology Research*.

[36] Touwendsida S. B., Bissoume S. B., Hadiza B. I. (2017). Isolation and characterization of enteropathogenic and enterotoxinogenic *Escherichia coli* from dairy products consumed in Burkina Faso. *African Journal of Microbiology Research*.

[37] Osaili T. M., Taani M., Al-Nabulsi A. A. (2013). Survival of escherichia coliO157: H7 during the manufacture and storage of fruit yogurt. *Journal of Food Safety*.

[38] Tohoyessou M. G., Mousse W., Sina H. (2020). Toxin production and resistance of *Staphylococcus* species isolated from fermented artisanal dairy products in Benin. *Journal of Pathogens*.

[39] Kagira J. M., Ngotho M., Mugo E., Kiplimo M., Maina N. (2022). Occurrence of antibiotic resistance in bacteria isolated from milk of dairy cows in small-holder farms in Juja sub-county, Kenya. *Asian Journal of Research in Animal and Veterinary Sciences*.

[40] Elmonir W., Abo-Remela E. M., Sobeih A. (2018). Public health risks of *Escherichia coli* and staphylococcus aureus in raw bovine milk sold in informal markets in Egypt. *The Journal of Infection in Developing Countries*.

[41] Okoko I. M., Maina N., Kiboi D., Kagira J. (2020). *β*-lactam resistance in bacteria associated with subclinical mastitis in goats in Thika sub-county, Kenya. *Veterinary World*.

[42] Sandhu R., Dahiya S., Sayal P. (2016). Evaluation of multiple antibiotic resistance (MAR) index and doxycycline susceptibility of *Acinetobacter* species among inpatients. *Indian Journal of Microbiology Research*.

[43] O’neill J. (2016). Tackling drug-resistant infections globally: final report and recommendations. *Review on Antimicrobial Resistance*.

[44] Anjum M. F., Marco-Jimenez F., Duncan D., Marín C., Smith R. P., Evans S. J. (2019). Livestock-associatedmethicillin-resistant*Staphylococcus aureus* from animals and animal products in the UK. *Frontiers in Microbiology*.

[45] Lee J., Jang K., Lee S. (2018). Prevalence of foodborne pathogens on cheeses made from farmstead milk processing plants in Korea. *Food Engineering Progress*.

[46] Arciola C. R., An Y. H., Campoccia D., Donati M. E., Montanaro L. (2005). Etiology of implant orthopedic infections: a survey on 1027 clinical isolates. *The International Journal of Artificial Organs*.

[47] Abid M., Bendahou A., Lebbadi M., Ennanei L., Essadqui F. Z. (2008). Characterization of *Staphylococcus* species isolated from raw milk and milk products (lben and jben) in North Morocco. *Journal of Infection in Developing Countries*.

[48] Veras J. F., do Carmo L. S., Tong L. C. (2008). A study of the enterotoxigenicity of coagulase-negative and coagulase-positive staphylococcal isolates from food poisoning outbreaks in Minas Gerais, Brazil. *International Journal of Infectious Diseases*.

[49] Meshref A. M. S. (2013). Bacteriological quality and safety of raw cow’s milk and fresh cream. *Slovenian Veterinary Research*.

[50] Baylis C. L. (2009). Raw milk and raw milk cheeses as vehicles for infection by verocytotoxin-producing*Escherichia coli*. *International Journal of Dairy Technology*.

[51] Tarr P. I., Gordon C. A., Chandler W. L. (2005). Shiga-toxin-producing*Escherichia coli* and haemolytic uraemic syndrome. *The Lancet*.

